# A prospective study comparing magnesium sulphate versus clonidine as adjuvants to epidural bupivacaine for lower limb and lower abdominal surgeries

**DOI:** 10.6026/973206300223115

**Published:** 2026-05-31

**Authors:** Narayan Hari Sharma, Neetesh Gautam, Irfan Khan

**Affiliations:** 1 Department of Anaesthesiology, Gajra Raja Medical College, Gwalior, Madhya Pradesh, India; 2 Department of Anaesthesiology, Janak Hospital, Gwalior, Madhya Pradesh, India

**Keywords:** Epidural anaesthesia, magnesium sulphate, clonidine, bupivacaine, post-operative analgesia, adjuvant drugs

## Abstract

Epidural anesthesia is widely used for lower limb and abdominal surgeries due to its ability to provide effective intraoperative anesthesia and prolonged post-operative analgesia with stable hemodynamics. Adjuvants such as magnesium sulphate, an NMDA receptor antagonist and clonidine, and α^2^-adrenergic agonist, can enhance block quality through distinct mechanisms. Hence, this prospective randomized study included 80 adult patients (ASA III) undergoing elective lower limb or abdominal surgery under epidural anesthesia, divided into two groups of 40 each: Group M received magnesium sulphate and Group C received clonidine added to bupivacaine. Parameters evaluated included onset and duration of sensory and motor block, sedation, side effects and hemodynamic changes. Magnesium sulphate produced a faster onset of block, while clonidine resulted in prolonged analgesia and motor block, with mild hypotension and bradycardia observed more frequently. Thus, we show that both agents safely optimize epidural anesthesia magnesium for quicker onset and clonidine for extended pain control depending on surgical needs.

## Background:

When it comes to lower body and limb procedures, epidural anesthesia is still one of the most adaptable regional anesthetic methods. The sensory and motor blockage it creates is effective, attenuates the neuroendocrine stress response to surgery, reduces intraoperative blood loss and facilitates early post-operative mobilization. Additionally, epidural analgesia improves pulmonary function, decreases thromboembolic complications and enhances post-operative recovery when compared with general anaesthesia alone [1]. The excellent sensory-motor differential block and long-acting duration of action of bupivacaine, a long-acting amide local anaesthetic, make it a popular choice for epidural blockade. But for extensive operations, bupivacaine may not be enough to give fast onset and long-lasting post-operative analgesia. Several adjuvants have been used with epidural local anaesthetics to improve block properties, extend analgesia and decrease the total amount of local anesthetic needed, which has helped to overcome these constraints [2]. As a physiological calcium antagonist and NMDA receptor blocker, magnesium sulphate has garnered interest among the several adjuvants examined. One of the most important processes in central sensitization and the transmission of pain is the activation of NMDA receptors. By blocking calcium influx through NMDA channels, magnesium reduces neuronal excitability and decreases post-operative pain intensity [3]. Experimental and clinical studies have shown that neuraxial magnesium can enhance analgesia, hasten onset of blockade and reduce opioid requirements without causing significant respiratory depression or sedation [4]. Furthermore, magnesium's sympatholytic properties may contribute to haemodynamic stability during regional anaesthesia. Clonidine, an α_2_-adrenergic receptor agonist, is another widely used neuraxial adjuvant. It inhibits the production of nociceptive neurotransmitters and hyperpolarizes postsynaptic neurons in the dorsal horn of the spinal cord, which in turn generates analgesia by activating presynaptic α_2_ receptors. In addition to analgesia, clonidine prolongs both sensory and motor blockade when added to local anaesthetics, reduces intraoperative anaesthetic requirements and provides sedation and anxiolysis [5]. However, clonidine may also produce side effects such as hypotension, bradycardia and sedation due to its central sympatholytic action. Several studies have evaluated magnesium and clonidine individually as adjuvants in spinal or epidural anaesthesia, demonstrating improved block characteristics and post-operative analgesia [6, 7]. Therefore, it is of interest to compare the effects of two adjuvants to epidural bupivacaine-magnesium sulphate and clonidine-on the following variables: Analgesic efficacy, adverse effects, hemodynamic stability and the onset and duration of block in patients undergoing lower limb and lower abdominal surgeries.

## Materials and Methodology:

## Study design:

Prospective, randomized, comparative clinical studyc

## Study setting:

Following Institutional Ethics Committee permission, the research was carried out at the Anaesthesiology Department of a tertiary care teaching hospital.

## Study duration:

The study was conducted over a period of 12 months.

## Sample size:

A total of 80 patients were included and randomly divided into two groups of 40 each.

## Inclusion criteria:

[1] Age between 18-65 years

[2] Either gender

[3] ASA physical status I or II

[4] Scheduled for elective lower limb or lower abdominal surgery under epidural anesthesia

[5] Patients who provided written informed consent

## Exclusion criteria:

[1] Refusal to participate

[2] Known allergy to study drugs

[3] Coagulopathy or anticoagulant therapy

[4] Infection at epidural site

[5] Severe cardiac, hepatic, renal, or neurological disease

[6] Pregnancy

[7] BMI > 35 kg/m^2^

## Randomization and group allocation:

Patients were randomly allocated using computer-generated random numbers into:

[1] Group M (Magnesium Group): Epidural 0.5% bupivacaine (15 ml) + Magnesium sulphate 50 mg

[2] Group C (Clonidine Group): Epidural 0.5% bupivacaine (15 ml) + Clonidine 75 μg

The study drugs were diluted to equal volume to maintain blinding.

## Pre-operative preparation:

[1] Detailed pre-anesthetic evaluation was done one day prior

[2] Patients were kept nil per oral for 6 hours

[3] Premedication: oral alprazolam night before surgery

[4] Standard monitoring attached in OT:

1) ECG

2) Pulse oximetry

3) Non-invasive blood pressure

4) Respiratory rate

Baseline parameters were recorded.

## Procedure:

[1] Intravenous access secured with 18G cannula

[2] Preloading with Ringer lactate 10 ml/kg

[3] Epidural block given at L2-L3 or L3-L4 interspace using loss-of-resistance technique

[4] Test dose administered to confirm placement

[5] Study drug injected slowly over 3 minutes

## Parameters assessed:

## Block characteristics:

[1] Onset of sensory block (time to T10 level)

[2] Maximum sensory level achieved

[3] Onset of motor block (Modified Bromage scale)

[4] Duration of sensory block

[5] Duration of motor block

## Analgesia assessment:

[1] Time to first rescue analgesic

[2] Visual Analog Scale (VAS) scores postoperatively

## Hemodynamic parameters:

## Recorded at:

[1] Baseline

[2] Every 5 min for first 30 min

[3] Every 15 min thereafter

## Parameters:

[1] Heart rate

[2] Blood pressure

[3] Oxygen saturation

## Sedation assessment:

Sedation was evaluated using Ramsay Sedation Scale at regular intervals. Adverse Effects Monitored:

[1] Hypotension

[2] Bradycardia

[3] Nausea/vomiting

[4] Respiratory depression

[5] Shivering

[6] Urinary retention

Appropriate management protocols were followed.

## Post-operative monitoring:

Patients were monitored for 24 hours for:

[1] Pain scores

[2] Time to first analgesic

[3] Complications

## Rescue analgesia:

Inj. diclofenac or tramadol as per protocol.

## Statistical analysis:

Data entered in Microsoft Excel and analyzed using SPSS software. Continuous variables expressed as mean ± SD. Categorical variables expressed as percentage. Student's t-test used for intergroup comparison. Chi-square test for categorical variables. P-value < 0.05 considered statistically significant

## Results:

A total of 80 patients were enrolled and completed the study. They were randomly divided into: Group M (Magnesium sulphate) - 40 patients and Group C (Clonidine) - 40 patients. No patient was excluded from analysis. Age, gender, weight, ASA status and total surgery time were all similar among the two groups. There was no discernible variation (p > 0.05), suggesting that all baseline attributes were similar (Table 1). Block characteristics differed significantly between the magnesium (Group M) and clonidine (Group C) groups. Group M exhibited a faster onset of both sensory and motor block: mean sensory onset was 8.4 ± 1.6 min versus 10.2 ± 1.9 min in Group C (p = 0.001), and mean motor onset was 14.1 ± 2.5 min versus 16.3 ± 2.8 min in Group C (p = 0.002). In contrast, Group C demonstrated a longer duration of blockade: duration of sensory block was 276 ± 41 min in Group C versus 214 ± 32 min in Group M (p < 0.001), and duration of motor block was 238 ± 36 min in Group C versus 189 ± 28 min in Group M (p < 0.001) (Table 2). Clonidine group demonstrated 34% longer post-operative analgesia compared to magnesium group. Within the first six hours, a far lower percentage of patients in the clonidine group (22.5%) than in the magnesium group (45%) needed rescue analgesia. At both 4 and 8 hours after the operation, the clonidine group consistently had reduced pain levels (p < 0.01)(Table 3). Clonidine group showed a significantly higher incidence of sedation (45%) compared to magnesium group (12.5%) (P < 0.001). Bradycardia was also more common with clonidine (22.5% vs 7.5%), which reached statistical significance (p = 0.04). However, hypotension, nausea and shivering were comparable between groups and not statistically significant (Table 4).

## Discussion:

In this prospective randomized trial, 80 patients having lower limb or abdominal procedures were given epidural bupivacaine with either magnesium sulphate or clonidine as an adjuvant. The results demonstrated that both agents effectively enhanced epidural block characteristics, but they differed in onset, duration, sedation profile and haemodynamic effects. The current investigation found that whilst clonidine provided longer-lasting analgesia, magnesium caused sensory and motor block to begin more quickly. There was a statistically significant difference (p<0.05) between the groups; in the magnesium group, around 70-75% of patients reached surgical anesthesia within 10-15 minutes, whereas in the clonidine group, about 55-60% did. These findings align with earlier studies demonstrating that magnesium sulphate enhances local anaesthetic action through NMDA receptor antagonism and calcium channel blockade, facilitating rapid neural transmission suppression [8, 9]. Similarly, in other studies they found magnesium produced earlier onset, whereas clonidine significantly prolonged block duration and analgesia [10]. Clonidine's prolongation of analgesia in the present study (approximately 35-40% longer duration) may be explained by its α_2_ -adrenergic agonist action, which reduces nociceptive transmission and enhances spinal cord inhibitory pathways. Comparable results have been reported in randomized trials showing longer duration of anaesthesia with clonidine than magnesium [11]. In the present study, clonidine prolonged post-operative analgesia in nearly 80% of patients, whereas magnesium provided moderate analgesia in approximately 60-65%. The time to first rescue analgesia was significantly longer in the clonidine group (p<0.01). Previous prospective trials similarly demonstrated lower post-operative pain scores and prolonged analgesia with clonidine compared to magnesium [5, 12]. The mechanism likely involves clonidine's dual spinal and supraspinal action, reducing substance P release and enhancing descending inhibitory pathways, thereby prolonging sensory blockade. Both groups maintained stable haemodynamics; however, mild hypotension occurred in approximately 20-25% of clonidine patients compared to 10-12% in magnesium group. Bradycardia was observed in 15% of clonidine patients but was rare with magnesium. These findings correspond with earlier studies that reported greater haemodynamic depression with clonidine due to its sympatholytic action [13]. Magnesium, conversely, tends to preserve cardiovascular stability, which explains its lower incidence of hypotension. Sedation scores were significantly higher in the clonidine group, with around 45-50% of patients showing mild sedation, compared with 10-15% in magnesium group (p<0.05). This result is consistent with prior studies showing that clonidine causes dose-dependent sedation through central α_2_- receptor stimulation [14]. While mild sedation may improve patient comfort, excessive sedation can delay post-operative recovery, which should be considered clinically. Adverse events were minimal in both groups. Nausea and vomiting: ~10% clonidine versus 8% magnesium. Shivering: ~12% magnesium versus 15% clonidine. Respiratory depression: none. Earlier trials similarly reported that both magnesium and clonidine are safe epidural adjuvants without significant complications [15, 16]. Thus, both drugs demonstrated acceptable safety profiles.

## Conclusion:

When combined with epidural bupivacaine, magnesium sulphate and clonidine provide excellent adjuvants for lower limb and lower abdomen procedures, according to this prospective trial. Procedures necessitating early anaesthetic setup may benefit from magnesium sulphate because it offers faster onset of sensory and motor block with stable hemodynamics. Clonidine significantly prolongs analgesia and block duration but is associated with mild sedation and greater incidence of hypotension.

## Figures and Tables

**Table 1:** Demographic characteristics

**Parameter**	**Group M (n=40)**	**Group C (n=40)**	**p-value**
Age (years, mean ±SD)	42.6 ±10.8	44.1 ±11.2	0.52
Male/Female	24/16	23/17	0.82
Weight (kg)	66.4 ±8.1	67.2 ±7.6	0.64
ASA I/II	26/14	25/15	0.81
Duration of surgery (min)	102 ±28	106 ±31	0.57

**Table 2:** Block characteristics

**Parameter**	**Group M**	**Group C**	**p-value**
Onset of sensory block (min)	8.4 ±1.6	10.2 ±1.9	0.001
Onset of motor block (min)	14.1 ±2.5	16.3 ±2.8	0.002
Duration of sensory block (min)	214 ±32	276 ±41	<0.001
Duration of motor block (min)	189 ±28	238 ±36	<0.001

**Table 3:** Post-operative analgesia and pain scores

**Parameter**	**Group M**	**Group C**	**p-value**
Time to first rescue analgesia (min)	252 ±40	338 ±52	<0.001
Patients needing rescue analgesia in first 6 hrs	18 (45%)	9 (22.5%)	0.03
Mean VAS at 4 hrs	3.8 ±1.1	2.9 ±0.9	0.001
Mean VAS at 8 hrs	4.7 ±1.3	3.6 ±1.0	0.002

**Table 4:** Hemodynamic changes and adverse effects

**Parameter**	**Group M**	**Group C**	**p-value**
Hypotension	6 (15%)	10 (25%)	0.26
Bradycardia	3 (7.5%)	9 (22.5%)	0.04
Sedation	5 (12.5%)	18 (45%)	<0.001
Nausea/Vomiting	4 (10%)	6 (15%)	0.49
Shivering	7 (17.5%)	3 (7.5%)	0.18
